# Enhanced(*R*)-2-(4-Hydroxyphenoxy)Propionic Acid Production by *Beauveria bassiana*: Optimization of Culture Medium and H_2_O_2_ Supplement under Static Cultivation

**DOI:** 10.4014/jmb.2002.02046

**Published:** 2020-05-20

**Authors:** Hai-Feng Hu, Hai-Yan Zhou, Xian-Lin Wang, Yuan-Shan Wang, Ya-Ping Xue, Yu-Guo Zheng

**Affiliations:** 1 Key Laboratory of Bioorganic Synthesis of Zhejiang Province, College of Biotechnology and Bioengineering, Zhejiang University of Technology, Hangzhou 310014, P.R. China; 2 Engineering Research Center of Bioconversion and Biopurification of Ministry of Education, Zhejiang University of Technology, Hangzhou 310014, P.R. China; 3 The National and Local Joint Engineering Research Center for Biomanufacturing of Chiral Chemicals, Zhejiang University of Technology, Hangzhou 310014, P.R. China

**Keywords:** (*R*)-2-(4-hydroxyphenoxy)propionic acid, optimization, reactive oxygen species, static cultivation, *Beauveria bassiana*

## Abstract

(*R*)-2-(4-hydroxyphenoxy)propionic acid (HPOPA) is a key intermediate for the preparation of aryloxyphenoxypropionic acid herbicides (*R*-isomer). In order to improve the HPOPA production from the substrate (*R*)-2-phenoxypropionic acid (POPA) with *Beauveria bassiana* CCN-A7, static cultivation and H_2_O_2_ addition were attempted and found to be conducive to the task at hand. This is the first report on HPOPA production under static cultivation and reactive oxygen species (ROS) induction. On this premise, the cultivation conditions and fermentation medium compositions were optimized. As a result, the optimal carbon source, organic nitrogen source, and inorganic nitrogen source were determined to be glucose, peptone, and ammonium sulfate, respectively. The optimal inoculum size and fermentation temperature were 13.3% and 28°C, respectively. The significant factors including glucose, peptone, and H_2_O_2_, identified based on Plackett-Burman design, were further optimized through Central Composite Design (CCD). The optimal concentrations were as follows: glucose 38.81 g/l, peptone 7.28 g/l, and H_2_O_2_ 1.08 g/l/100 ml. Under the optimized conditions, HPOPA titer was improved from 9.60 g/l to 19.53 g/l, representing an increase of 2.03- fold. The results obtained in this work will provide novel strategies for improving the biosynthesis of hydroxy aromatics.

## Introduction

Aryloxyphenoxypropionic acid-type herbicide is one of the best kinds of herbicides. It is non-toxic to crops, has a long expiration date, and degrades easily in the soil [1, 2]. Actually, two isomers (*R*- and *S*-isomer) exist in aryloxyphenoxypropionic acid-type herbicide [3], and the *R*-type herbicide has proved to be much more effective than the *S*-type one [4]. Therefore, the production of enantiomerically pure *R*-type herbicides is more promising. At present, the enantiomerically pure aryloxyphenoxypropionic acid-type herbicides (*R*-isomer) such as fenoxaprop-*P*-ethyl, clodinafop-propargyl, propaquizafop, and metamifop, etc. [5, 6] are mainly synthesized from (*R*)-2-(4-hydroxyphenoxy)propionic acid (HPOPA). As a key intermediate, HPOPA has been in increasing demand in the herbicide industry and HPOPA production has drawn more attention in recent years [7].

As one of the most common biocatalysts, entomopathogenic fungus *Beauveria bassiana* has multiple applications, especially in the hydroxylation of saturated and aromatic carbon atoms, Baeyer-Villiger/sulfide oxidation, keto-alcohol/alkene redox reaction, heteroatom dealkylation and epoxide, ester hydrolysis and glucosidation [8-12]. In our earlier study, a wild-type *B. bassiana* ZJB16001 [7, 13] capable of hydroxylating (*R*)-2- phenoxypropionic acid (POPA) at C-4 site into HPOPA, was obtained from environmental samples and the derivative strain *B. bassiana* CCN-A7 was obtained after multi-round mutagenesis. The HPOPA titer of *B. bassiana* CCN-A7 was 9.6 g/l after agitated fermentation for 7 d in 30-ml medium containing 20 g/l POPA. To improve the HPOPA production of *B. bassiana* CCN-A7, the optimization of the nutrition components and culture conditions was performed in this work. In recent years, static cultivation methods were successfully applied in the production of various biochemicals, as for example, ganoderic acid biosynthesis by *Ganoderma lucidum*, cordycepin production by *Cordyceps militaris*, and bacterial nanocellulose production [14-16]. Herein, we attempted to investigate the effect of agitation speeds on HPOPA production by *B. bassiana* CCN-A7, and found that static cultivation was a more suitable fermentation mode for HPOPA production compared with the traditional agitated cultivation.

In many organisms, reactive oxygen species (ROS) are highly reactive molecules mainly including superoxide radicals (O_2_^−^), hydrogen peroxide (H_2_O_2_), and hydroxyl radicals (HO^−^), all of which function in regulating various physiological processes [12,17-19]. There has been increasing evidence that oxidative stress could cause the post- translational modification of enzymes and thus change enzymes activity rapidly and reversibly. As reported, in glycolysis pathway, the key enzyme glyceraldehyde-3-phosphate dehydrogenase (GAPDH) could be inactivated after exposure to various oxidant [20]. Therefore, it is expected that the targeted metabolites biosynthesis capacity of microbial species could be significantly enhanced when a reasonable ROS treatment is designed [21]. In this study, the influences of induced ROS on HPOPA production of *B. bassiana* CCN-A7 were investigated using an ROS generator (external H_2_O_2_). The external H_2_O_2_ was added into the fermentation medium of *B. bassiana* CCN- A7 and its concentration in the medium was optimized to improve HPOPA production. It is a novel strategy to enhance HPOPA biosynthesis via ROS induction.

In order to rapidly develop a practically feasible bioprocess, the comprehensive and systematical optimization of nutritional components and culture conditions is necessary [22]. Generally, medium optimization with the one-factor-at-a-time method is very laborious and time-consuming work, and the determination of suitable conditions is often not well guaranteed [23, 24]. The Plackett-Burman (PB) design is usually used as a preliminary optimization technique and provides unbiased estimation of all the variables by running only a few experiments [25]. Response Surface Method (RSM) is a collective statistical technique based on experiment designing, model building, evaluating the effects of factors, and finding the optimum conditions. RSM has become more popular than the conventional methods, mainly because the interaction among several variables can also be evaluated by mathematical modeling, when little information about the process is available [26-28]. In RSM, the most frequently used design of experiments (DOE) is Central Composite Design (CCD), in which slope-rotatability can be easily achieved. When it is difficult to extend the design region beyond the defined variable bounds, a face- centered CCD can be chosen [29].

The purpose of this work was to improve HPOPA production with *B. bassiana* CCN-A7 through optimization of the culture conditions under static cultivation and ROS induction. The effects of significant variables (medium components including H_2_O_2_ supplement) on HPOPA production were investigated through PB design and CCD. As a result, HPOPA production was improved significantly.

## Materials and Methods

### Chemicals

The HPOPA standard was purchased from J&K Chemical Ltd. (China). POPA was synthesized by Weifang Rainbow Chemical Co., Ltd. (China).

### Strain and Medium

*B. bassiana* CCN-A7, a derivative of *B. bassiana* ZJB16001 [7, 13] was used for HPOPA production in this study.

Potato dextrose agar medium (PDA) was composed of 200 g/l potato, 20 g/l glucose, and 20 g/l agar. Potato dextrose broth medium (PDB) was similar to PDA, except that agar was absent.

The initial fermentation medium was composed of 20 g/l glucose, 5 g/l yeast extract, 5 g/l (NH_4_)_2_SO_4_, 0.5 g/l MgSO_4_•7H_2_O, 0.05 g/l MnSO_4_•H_2_O, 1.5 g/l KH_2_PO_4_, 3.6 g/l K_2_HPO_4_•3H_2_O, 1 ml/l microelement solution (2 g/l FeSO_4_•7H_2_O, 100 mg/l ZnSO_4_•4H_2_O, 300 mg/l H_3_BO_3_, 200 mg/l C°Cl_2_•6H_2_O, 10 mg/l CuCl_2_•2H_2_O, 20 mg/l NiCl_2_•6H_2_O, 30 mg/l Na2MoO_4_•2H_2_O), and 20 g/l POPA, pH 6.8.

### Cultivation of *B. bassiana* CCN-A7 for HPOPA Production

The *B. bassiana* CCN-A7 was grown on PDA plates at 28°C for 7 d. A single colony was inoculated into a shake flask containing 30 ml PDB and grown at 28°C, 200 rpm for 3 d. Then, 1 ml seed broth of *B. bassiana* CCN-A7 was incubated into 30 ml autoclaved fermentation medium and cultured at 28°C for 7 d. In the case of H_2_O_2_ addition, at the cultivation time of 3 d, H_2_O_2_ was supplemented into the fermentation broth and the cultivation lasted for another 4 d.

### HPOPA, POPA and Biomass Measurements

HPOPA and POPA concentrations were measured through HPLC analysis as described previously [30]. In order to measure the dry cell weight (DCW), fermentation broth was centrifuged at 12,000 *g* for 5 min. The obtained biomass was washed twice with distilled water, and was dried at 65°C until constant weight. All measurements were carried out at least in triplicate.

### Agitated Cultivation and Static Cultivation for HPOPA Production

To compare the effect of agitated cultivation and static cultivation on HPOPA production, different agitation speeds 50 rpm, and 150 rpm were tested including static cultivation (0 rpm). The other culture conditions were the same as those mentioned above.

### Effect of H_2_O_2_ Supplementation on HPOPA Production

To investigate the effect of H_2_O_2_ supplementation on HPOPA production, 300 μl of H_2_O_2_ was added into the fermentation broth at cultivation time of 3 d and the cultivation lasted for another 4 d under static condition at 28°C.

### Selection of Carbon and Nitrogen Sources for HPOPA Production

**Effect of different carbon sources on HPOPA production**. In the fermentation medium, glucose was substituted with seven different carbon sources viz., fructose, lactose, maltose, sorbitol, soluble starch, glycerin, and sucrose. All carbon sources were used at 20 g/l and their effects on HPOPA production were singularly evaluated.

**Effect of different nitrogen sources on HPOPA production**. To study the effect of different nitrogen sources on HPOPA production, the organic nitrogen source yeast extract in the initial fermentation medium was replaced with the same concentration of beef extract, peptone, maize flour, peanut powder, and silkworm pupa meal, respectively. The inorganic nitrogen source ammonium sulfate was replaced with sodium nitrate, ammonium chloride, urea, ammonium acetate, sodium nitrite, ammonium formate, and ammonium dihydrogen phosphate, respectively. All nitrogen sources were used at 5 g/l and their effects on HPOPA production were singularly evaluated.

### PB Design and Analysis

To screen the significant factors, 9 variables including peptone, glucose, (NH_4_)_2_SO_4_, MnSO_4_•H_2_O, MgSO_4_•7H_2_O, K_2_HPO_4_•3H_2_O, KH_2_PO_4_, microelement solution, and H_2_O_2_ were selected and tested in a PB design experiment, which was conducted with a 9-factor-12-runs (Table S1). Each run was performed in triplicate. For statistical modeling, the first-order polynomial linear model as shown in Eq. (1) was employed.

(1) $$Y=\beta_{0}+\sum_{n=1}^{9}\beta_{n}x_{n}$$

where *Y* is the predicted response HPOPA titer (g/l), *β_0_* is the model intercept, *β_n_* is the linear coefficient, and *x_n_* is the coded level of the independent variable. The model for Eq. (1) was implemented using software Design Expert version 10.0.4 (Stat-Ease, USA). Significance level was set as *p*-value < 0.05. The significant independent variables were selected for further optimization based on CCD.

### Determination of the Center Point of Each Significant Variable for CCD

The most significant independent variables glucose, peptone, and H_2_O_2_ identified by PB experiment were selected for further optimization in CCD. To select the center point of each variable, glucose concentration was changed from 10 to 50 g/l, and peptone concentration was changed from 4 to 8 g/l, respectively. The effects of different concentrations of glucose (10-50 g/l) and peptone (4-8 g/l) as well as different amounts of H_2_O_2_ supplement (0-400 μl/30 ml medium) on HPOPA titer were examined, respectively. To select the optimal inoculum size and fermentation temperature for the following CCD experiment, the effects of different inoculum sizes varying from 3.3 to 16.7% and temperatures in the range of 24-32°C on HPOPA titer were tested, respectively.

### Central Composite Design and Analysis

A design of the experimental method using CCD packaged in Design Expert version 10.0.4 (Stat-Ease) was applied to improve the production of HPOPA. The most significant independent variables (glucose, peptone, and H_2_O_2_) identified by PB experiment were selected for further optimization. Each factor was assessed at five levels as shown in Table S2. The CCD-based experiment included 20 runs comprising 8 (2^3^) factorial points, 6 axial points, and 6 replicates at the center points. The dependent response was HPOPA production. The results were analyzed using analysis of variance (ANOVA) through evaluation of the *F*-values and *p*-values and a significant model for HPOPA production was then established. The quality of the fit of the polynomial model was expressed by the determination coefficient *R*^2^. The objective function was to maximize the HPOPA production by optimizing the concentration/amount of glucose, peptone, and H_2_O_2_. The mathematical relationships between the response and the independent variables were described by a quadratic polynomial equation as given in Eq. (2):

(2) $$Y=\beta_{0}+\sum_{i=1}^{k}\beta_{i}x_{i}+\sum_{i=1}^{k}\beta_{i}x_{i}^{2}+\sum_{i=1}^{k}\sum_{i\neq j=1}^{k}\beta_{ij}x_{i}x_{j}$$

where *Y* refers to the predicted responses, *x_i_* and *x_j_* are independent variables, *β_0_* is the offset term, *β_i_*, *β_ii_*, and *β_ij_* are the linear, quadratic, and interactive coefficients, respectively, and *k* is the number of factors.

## Results and Discussion

### The Effect of Different Agitation Speeds on HPOPA Production

**Fig. 1:**
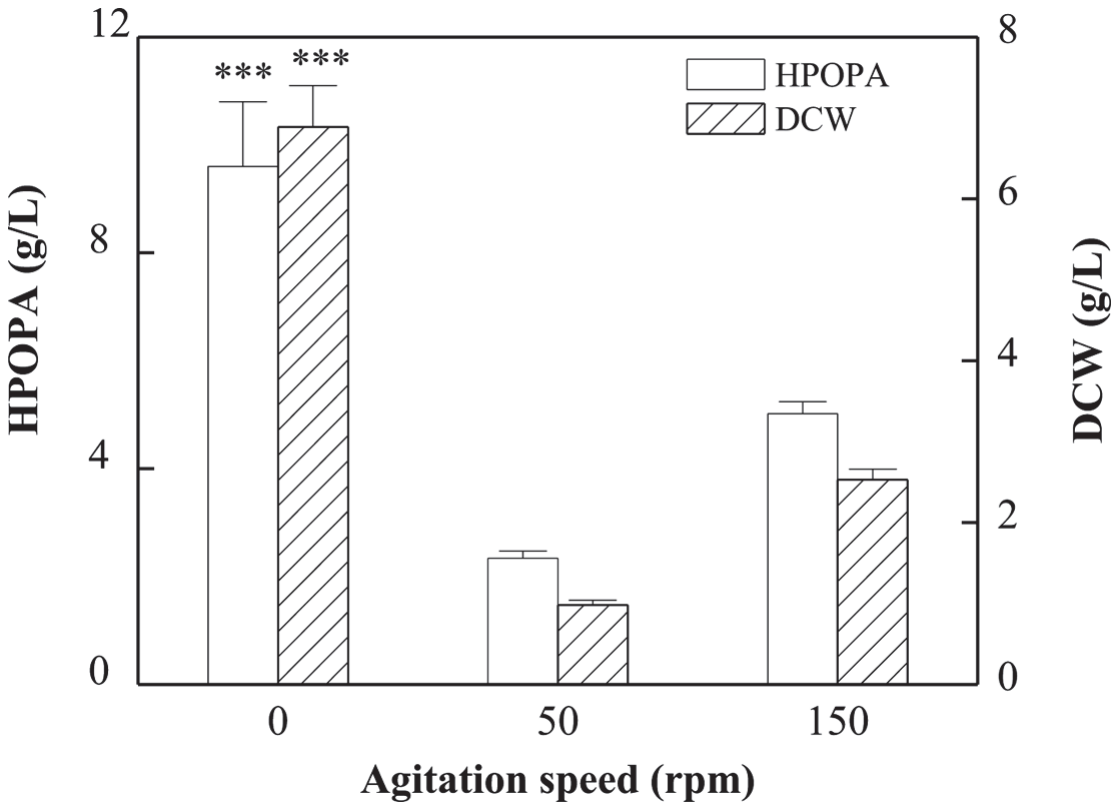
Effect of different agitation speeds on cell growth and HPOPA production. ***Extremely significant (the *p*-value < 0.001). The *P*-values were calculated between the relatively high levels and the level of the control (150 rpm).

The effect of different agitation speeds including 0, 50, and 150 rpm on cell growth and HPOPA production was investigated and the results were displayed in Fig. 1. It was found that the highest biomass was detected when the agitation speed was 0 rpm, that is, static cultivation (*p* = 0.0001). Furthermore, the highest HPOPA titer was also obtained under static condition (*p* = 0.0029). This indicated that, compared with agitated cultivation, static cultivation was more suitable for HPOPA production by *B. bassiana* CCN-A7. Different from the growth pattern in agitated culture, the cells grew adherently and the mycelia formed a layer till they completely covered the surface of the medium in static culture. Therefore, the oxygen availability in the overlay fluid above the attached cells was relatively higher than that in the liquid submerged fermentation, which might be the cause for enhanced HPOPA biosynthesis. In addition, static culture could also avoid the damage to the hyphae caused by shear stress in agitated culture. Therefore, static cultivation was applied in the following experiments.

### Effect of H_2_O_2_ Supplementation on HPOPA Production

**Fig. 2:**
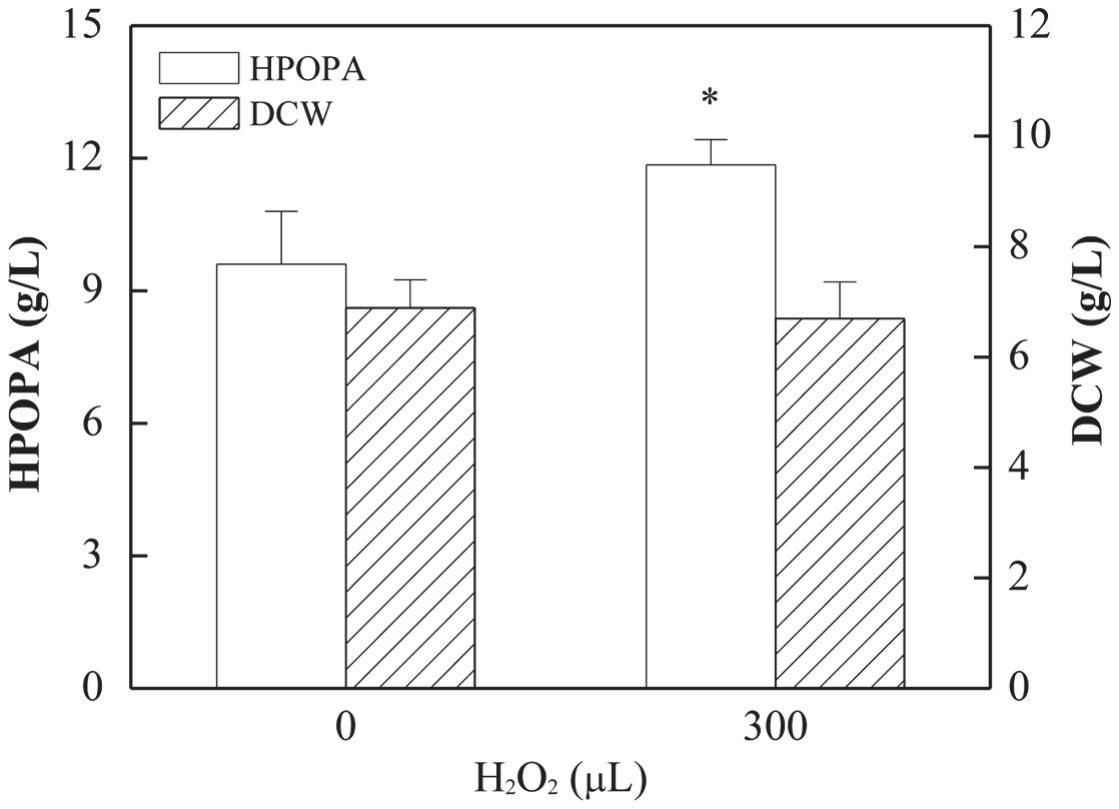
Effect of H_2_O_2_ addition on cell growth and HPOPA production. *Significant (the *p*-value < 0.05). The *p*-values were calculated between the relatively high levels and the level of the control (0 μl H_2_O_2_ addition).

As shown in Fig. 2, compared with the control (without H_2_O_2_ addition), H_2_O_2_ supplementation (300 μl) resulted in 1.23-fold higher HPOPA titer (*p* = 0.0390). This indicated that H_2_O_2_ supplement improved the HPOPA production by *B. bassiana* CCN-A7. It was speculated that the enzyme responsible for the synthesis of HPOPA may be a type of peroxidase [31], the activity of which is dependent on the presence of H_2_O_2_. This is the first report on the effect of H_2_O_2_ on enhancement of HPOPA biosynthesis. Therefore, ROS induction can be a novel strategy for improving HPOPA production. Furthermore, this finding provided some important references for investigating the mechanism of HPOPA synthesis by *B. bassiana*.

### Selection of Suitable Carbon and Nitrogen Sources for HPOPA Production

**Fig. 3:**
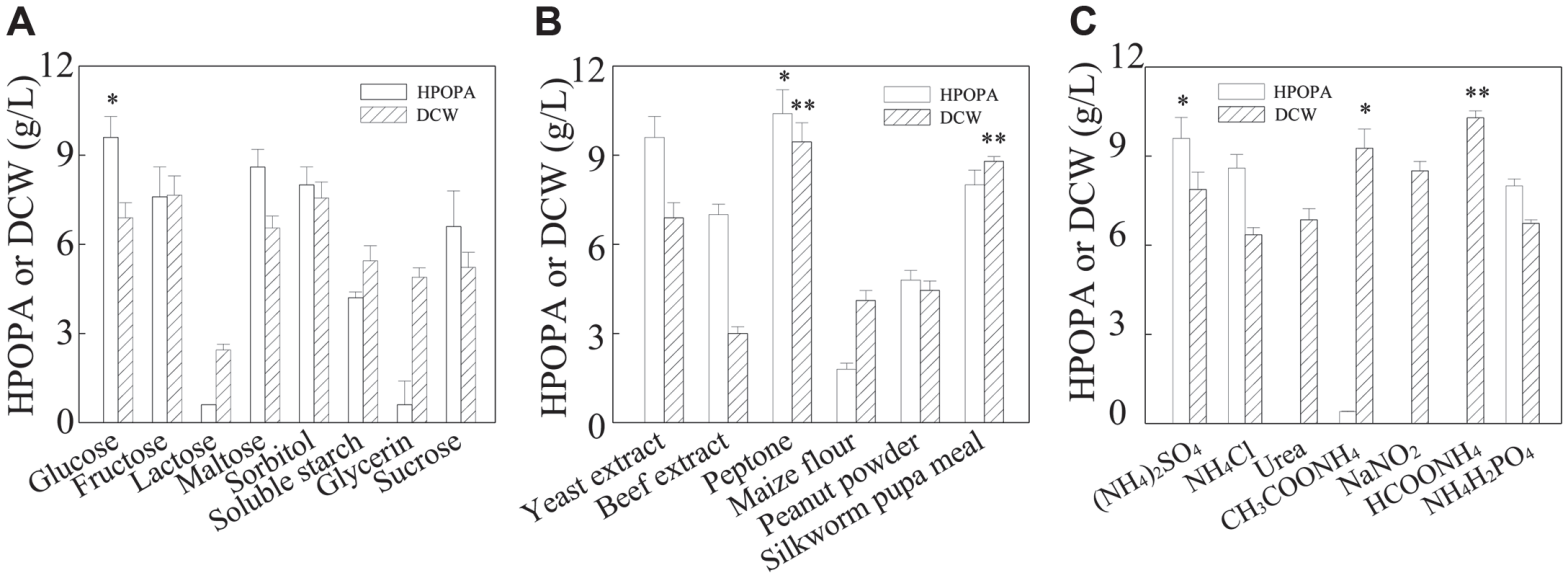
Effect of different carbon and nitrogen sources on cell growth and HPOPA production. (**A**) Carbon sources (the control was glucose); (**B**) Organic nitrogen sources (the control was yeast extract); (**C**) Inorganic nitrogen sources (the control was ammonium sulfate). ***Extremely significant (the *p*-value < 0.001); **Highly significant (the *p*-value < 0.01); *Significant (the *p*-value < 0.05). The *P*-values were calculated between the relatively high levels and the level of the control in each experiment.

Among the different carbon sources tested, the utilization of fructose and glucose leads to the maximum biomass and HPOPA titer, respectively (Fig. 3A). Maltose and sorbitol also supported a comparable level of HPOPA production. While HPOPA production was lower in the case of lactose and glycerin, probably due to the fact that lactose and glycerin were not efficiently used by *B. bassiana* CCN-A7 for HPOPA production. It should be noted that, although fructose, sorbitol, and glucose supported a comparable level of cell growth, the maximum HPOPA production (9.60 ± 1.20 g/l) was achieved when glucose was used (*p* = 0.0179, glucose vs. maltose), followed in sequence by maltose, sorbitol, fructose, sucrose, soluble starch, glycerin, and lactose with regard to the product titer from high to low. Therefore, glucose was selected for further optimization studies.

As shown in Fig. 3B, among the various organic nitrogen sources, beef extract and urea could not well support the growth of *B. bassiana* CCN-A7. By contrast, peptone caused the highest biomass (9.45 ± 1.72 g/l) (*p* = 0.0425, peptone vs. yeast extract) and HPOPA titer (10.40 ± 1.20 g/l) (*p* = 0.0056, peptone vs. yeast extract), which was followed from high to low by that of yeast extract, silkworm pupa meal, beef extract, peanut powder, and maize flour. Peptone was thus regarded to be the optimum nitrogen source for HPOPA production and was used in the following studies.

Fig. 3C showed that, among the various inorganic nitrogen sources, urea, sodium nitrite, and ammonium formate did not support the HPOPA production by *B. bassiana* CCN-A7; ammonium formate and ammonium sulfate led to the highest biomass (8.11 ± 1.39 g/l) and HPOPA production (9.60 ± 1.20 g/l), respectively. The HPOPA production reached the highest level when ammonium sulfate was used (*p* = 0.0452, ammonium sulfate vs. ammonium chloride), followed from high to low by ammonium chloride, ammonium dihydrogen phosphate, ammonium acetate, urea, sodium nitrite, and ammonium formate. Ammonium sulfate was thus recommended to be the most suitable inorganic nitrogen source for HPOPA production and was used in the following studies.

### PB Design and Analysis for HPOPA Production

The results of the fermentation medium optimization under the PB design for HPOPA production were shown in Table S1. After fitting a first order polynomial model, the coefficient of determination (*R*^2^) was 0.9888 (Table 1), which indicated a good fit. As shown in Table 1, three factors including glucose (*p* = 0.0178), peptone (*p* = 0.0196), and H_2_O_2_ (*p* = 0.0189) were found to have significant effects on HPOPA production. Therefore, they were selected for the following CCD-based optimization experiments.

### Determination of the Center Point of Each Significant Variable

**Fig. 4:**
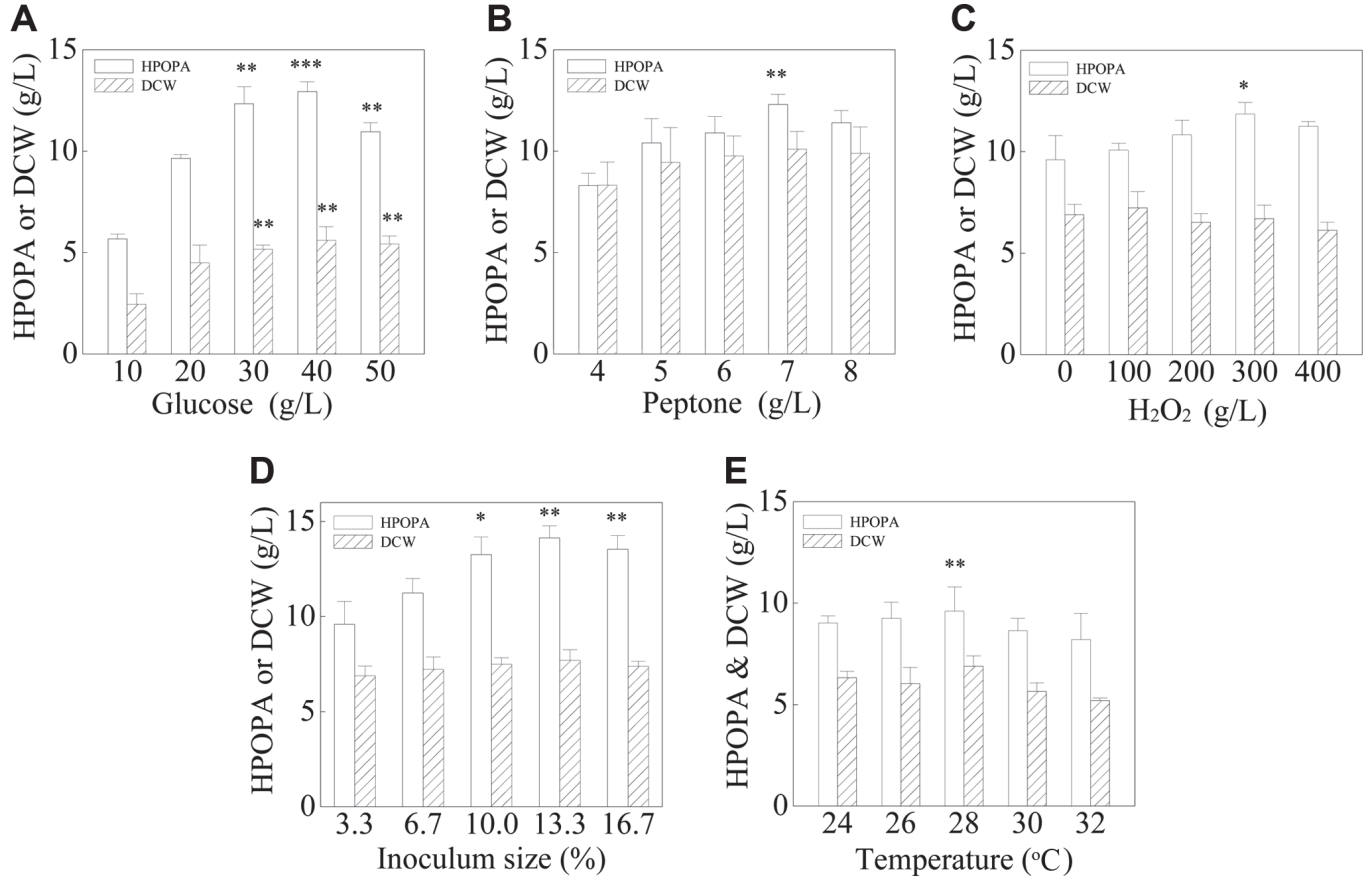
Effect of different conditions on cell growth and HPOPA production. (**A**) Glucose concentration (the control was 20 g/l); (**B**) Peptone concentration (the control was 5 g/l); (**C**) H_2_O_2_ addition amount (the control was 0 μl); (**D**) Inoculum size (the control was 3.3%); (**E**) Fermentation temperature (the control was 24°C). ***Extremely significant (the *p*- value < 0.001); **Highly significant (the *p*-value < 0.01); *Significant (the *p*-value < 0.05). The *p*-values were calculated between the relatively high levels and the level of the control in each experiment.

Prior to the CCD experiments, the center point of each significant variable identified by PB design and the optimum inoculum size and fermentation temperature were determined. From Figs. 4A-4B we can see that, the optimal concentrations of glucose and peptone for HPOPA production were 40 g/l (*p* = 0.0004) and 7 g/l (*p* = 0.0350), respectively. The optimal volume of H_2_O_2_ supplement was 300 μl/30 ml medium (*p* = 0.0390) (Fig. 4C). Higher H_2_O_2_ (400 μl) supplement was toxic to cell growth, and resulted in a decreased HPOPA production. The similar phenomenon was observed in other studies [32, 33]. The reason might be that some materials/energy were redistributed to eliminate the negative effects under ROS stress [34]. According to a previous report, under environmental stress, the cells could synthesize some antioxidant enzymes to clear the excessive ROS to maintain a stable level [35, 36].

As for the fermentation conditions, the highest HPOPA titers (14.14 ± 0.63 g/l and 9.60 ± 1.20 g/l) were obtained when the inoculum size was 13.3% (Fig. 4D) and at 28°C (Fig. 4E), respectively. Therefore, the optimum inoculum size and fermentation temperature were 13.3% (*p* = 0.0044) and 28°C (*p* = 0.0022), respectively, which were adopted in the following CCD experiment. The optimum concentrations of glucose/peptone and H_2_O_2_ supplement volume were 40 g/l, 7 g/l, and 300 μl/30 ml, respectively, which were used as the center points of CCD runs.

### Central Composite Design and Analysis for HPOPA Production

Experimental HPOPA production based on CCD was shown in Table S3. The parameters for estimation of the model and model fitting through fitting the experimental data using RSREG procedure with the ridge max option were shown in Table 2. A significant model (*p* < 0.0001) with non-significant lack of fit (*p* = 0.15) and a desirable *R*^2^ value of 0.9717 was developed. There are no interactions among these three variables. The analysis results also indicated that the model (Eq. 3) was adequate to fit the experimental data. The optimized level of each factor was 38.81 g/l of glucose, 7.28 g/l of peptone, 1.08 ml/100 ml of H_2_O_2_ and the predicted maximum HPOPA production was 19.21 g/l.

(3) $$\text{HPOPA (g/l) =}-14.42+0.49x_{1}+4.88x_{3}-1.75\times 10^{-3}x_{1}x_{2}-2.63\times 10^{-3}x_{1}x_{3}+0.036x_{2}x_{3}-6.07\times 10^{-3}x_{1}^{2}-0.40x_{2}^{2}-2.79x_{3}^{2}$$

**Fig. 5:**
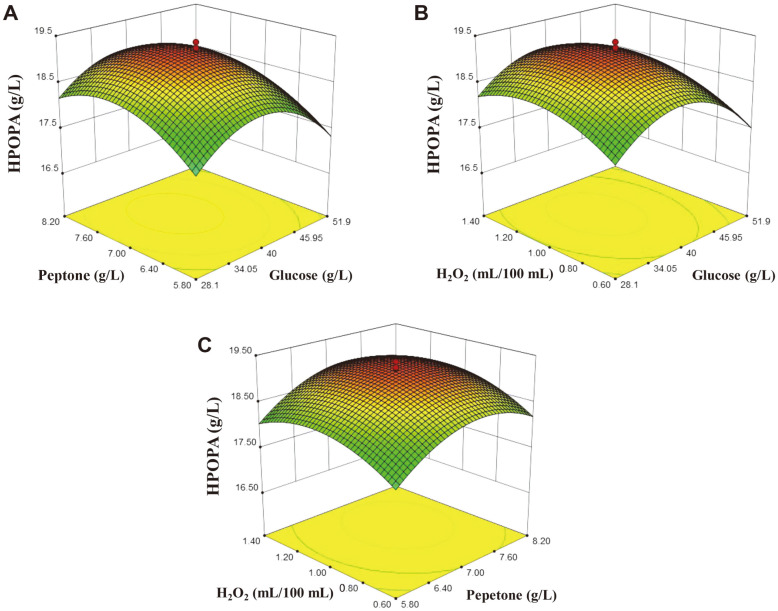
Response surface and contour plot exhibiting overall effects of multiple variables on HPOPA production. (**A**) Glucose and peptone (H_2_O_2_: 1 ml/100 ml); (**B**) Glucose and H_2_O_2_ (peptone: 7.0 g/l); (**C**) Peptone and H_2_O_2_ (glucose: 40 g/l).

The overall effects were illustrated in Fig. 5, in which, two factors were plotted as the independent variables and HPOPA titer was the dependent variable with the third variable constant at its optimum value. The response surface plots were in good consistency with the maximum HPOPA titer at the optimum condition. All the factors including glucose, peptone, and H_2_O_2_ exhibited a clear quadratic effect, which was in good agreement with the extremely significant effects (*p* < 0.0001) of quadratic terms as shown in Table 2. According to the *p-*values, the interaction effects between any two variables among glucose, peptone and H_2_O_2_ were not significant (*p* > 0.05), while the linear effects of glucose and H_2_O_2_ were significant (*p* < 0.05) and the linear effect of peptone was highly significant (*p* < 0.01). Noticeably, the quadratic effect of each variable was extremely significant (*p* < 0.001).

### Optimum Conditions and Validation

**Fig. 6:**
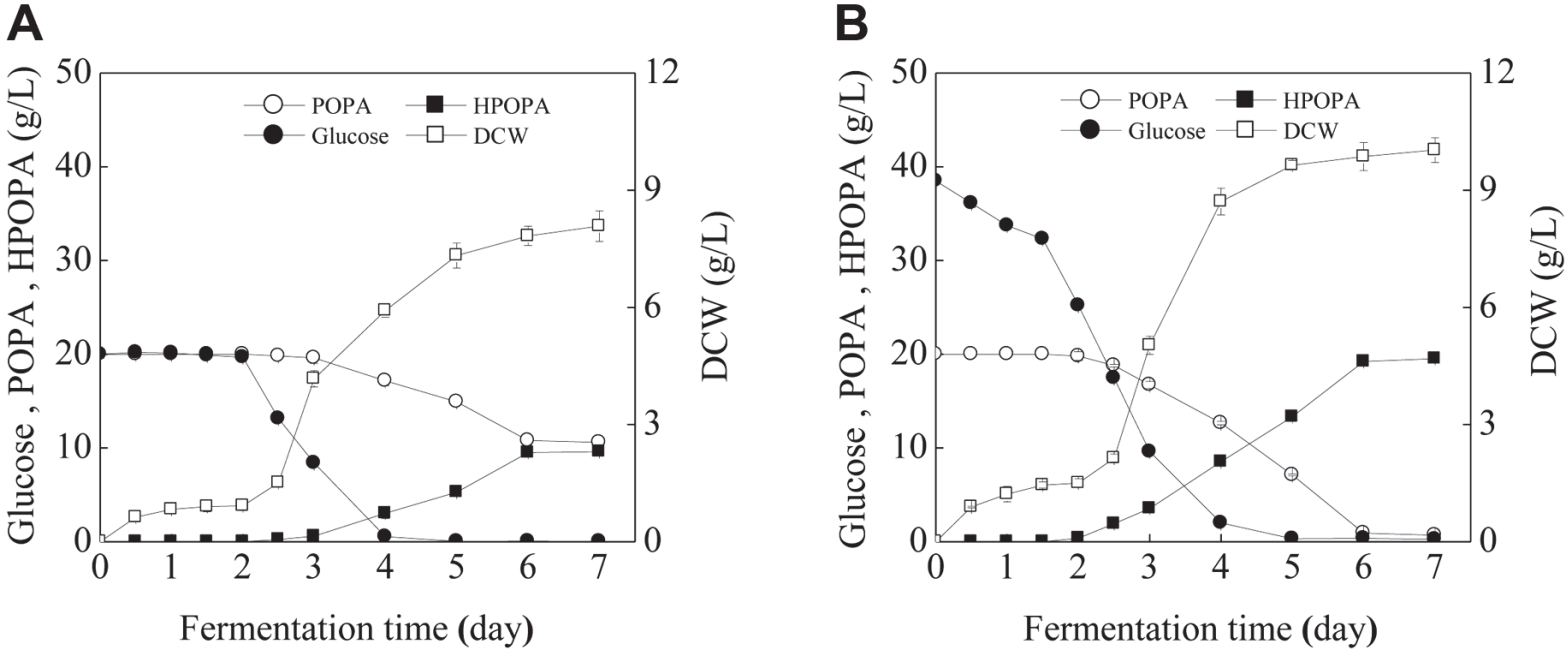
Fermentation curve of *B. bassiana* CCN-A7 with original fermentation medium (A) and optimized fermentation medium (B).

As a result, the optimal concentrations of glucose and peptone were 38.81 g/l and 7.28 g/l, respectively; and the optimal H_2_O_2_ supplement volume was 1.08 ml/100 ml. The predicted results were validated by experiments in triplicates. Meanwhile, the fermentation course was investigated, during which the parameters including POPA, HPOPA, glucose, and biomass at certain time intervals were measured. As shown in Figs. 6A-6B, the initial time of HPOPA production in optimal medium began earlier than that in the original medium and the entire fermentation time was shortened to 6 d from 7 d. In addition, with the optimal medium, glucose consumption started at 0.5 d, earlier than that of the original medium and glucose was exhausted at 5 d, later than the control.

Furthermore, at the end of the fermentation process, a higher HPOPA titer of 19.53 ± 0.29 g/l was obtained, representing a good agreement with the predicted value of 19.21 g/l.

In summary, static cultivation and ROS induction were adopted for improvement of HPOPA production by *B. bassiana* CCN-A7. In fermentation optimization, glucose, peptone, and ammonium sulfate were selected as the optimized carbon source, organic nitrogen source, and inorganic nitrogen source, respectively. Furthermore, it was found that the effects of glucose, peptone, and H_2_O_2_ were significant for HPOPA production through PB design. Subsequently, the concentrations/amounts of glucose, peptone, and H_2_O_2_ were optimized for improved HPOPA production based on CCD. As a result, a suitable level for each factor was determined: 38.81 g/l glucose, 7.28 g/l peptone, and 1.08 ml/100 ml H_2_O_2_. Under the optimized conditions, HPOPA titer was greatly improved from 9.60 g/l to 19.53 g/l, which was 2.03-fold higher compared with that of the initial fermentation medium. It should be highlighted that static cultivation was found to be more suitable for HPOPA production by *B. bassiana* CCN-A7 and ROS induction can improve HPOPA biosynthesis. These results obtained in this work may provide some new strategies to further improve HPOPA production.

## Supplemental Materials



Supplementary data for this paper are available on-line only at http://jmb.or.kr.

## Figures and Tables

**Table 1:** ANOVA for 12-run Plackett-Burman design.

Source De	grees of freedom Sum of squares (DF) (SS)	Mean square (MSE)	*F* value	*p*-value Prob > *F*	Significant
Model	9	283.66	31.52	19.56 0.0496	*
Peptone	1	79.77	79.77	49.51 0.0196	*
Glucose	1	88.13	88.13	54.70 0.0178	*
(NH_4_)_2_SO_4_	1	0.087	0.087	0.054 0.8381	
MnSO_4_•H_2_O	1	21.49	21.49	13.34 0.0675	
MgSO_4_•7H_2_O	1	10.38	10.38	6.44 0.1265	
K_2_HPO_4_•3H_2_O	1	2.133 × 10^-3^	2.133 × 10^-3^	1.324 × 10^-3^ 0.9743	
KH_2_PO_4_	1	0.012	0.012	7.468 × 10^-3^ 0.9390	
Microelement solution	1	0.78	0.78	0.48 0.5585	
H_2_O_2_	1	83.00	83.00	51.52 0.0189	*
Residual	2	3.22	1.61		
Total	11	286.88			
*R*-squared	0.9888				

Values of “Prob. > *F* ” less than 0.05 indicate that model terms are significant (*).

**Table 2:** ANOVA for CCD.

Source	DF	SS	MSE	*F* value	*p*-value Prob. > *F*	Significant
Model	9	17.21	1.91	38.17	< 0.0001	***
*x_1_* (glucose)	1	0.37	0.37	7.39	0.0216	*
*x_2_* (peptone)	1	0.89	0.89	17.71	0.0018	**
*x_3_* (H_2_O_2_)	1	0.42	0.42	8.48	0.0155	*
x_1_x_2_	1	5.000 × 10^-3^	5.000 × 10^-3^	0.100	0.7586	
x_1_x_3_	1	1.250 × 10^-3^	1.250 × 10^-3^	0.025	0.8776	
x_2_x_3_	1	2.450 × 10^-3^	2.450 × 10^-3^	0.049	0.8295	
$x_{1}^{2}$	1	10.63	10.63	212.16	< 0.0001	***
$x_{2}^{2}$	1	4.67	4.67	93.11	< 0.0001	***
$x_{3}^{2}$	1	2.88	2.88	57.47	< 0.0001	***
Residual	10	0.50	0.050	
Lack of fit	5	0.36	0.073	2.66	0.1533	Not significant
Pure error	5	0.14	0.027			
Cor total	19	17.71				
Std. Dev.	0.22					
Mean	17.87					
C.V. %	1.25					
PRESS	2.96					
*R*-squared	0.9717					
Adj *R*-squared	0.9463					
Pred *R*-squared	0.8329					
Adeq precision	17.098					

Values of “Prob. > *F*” less than 0.001, 0.01, and 0.05 indicate that model terms are extremely significant (***), highly significant (**), and significant *), respectively.
