# Supplementary material for: Enhanced(R)-2-(4-Hydroxyphenoxy)Propionic Acid Production by Beauveria bassiana: Optimization of Culture Medium and H2O2 Supplement under Static Cultivation
Source: J Microbiol Biotechnol. 2020 May 20;30(8):1252–60. doi: 10.4014/jmb.2002.02046 (PMC9728401; doi:10.4014/jmb.2002.02046)
Supplement: Supplementary file 1 [file JMB-30-8-1252-supple.pdf]

J. Microbiol. Biotechnol.
https://doi.org/10.4014/jmb.2002.02046

Supplementary Table S1. HPOPA production for 12 runs with 9 factors in Plackett-Burman design

Run	Peptone (g/L)	Glucose (g/L)	$(NH_4)_2SO_4$ (g/L)	$MnSO_4·H_2O$ (g/L)	$MgSO_4·7H_2O$ (g/L)	$K_2HPO_4·3H_2O$ (g/L)	KH_2PO_4 (g/L)	Microelement solution (mL/100 mL)	H_2O_2 (mL/100 mL)	HPOPA (g/L)
1	8 (1)	30 (1)	3 (-1)	0.20 (1)	2.0 (1)	7.2 (1)	1.5 (-1)	0.1 (-1)	0.5 (-1)	8.15 ± 0.13
2	3 (-1)	5 (-1)	8 (1)	0.05 (-1)	2.0 (1)	7.2 (1)	1.5 (-1)	0.5 (1)	2 (1)	4.83 ± 0.21
3	3 (-1)	30 (1)	8 (1)	0.05 (-1)	2.0 (1)	7.2 (1)	3.0 (1)	0.1 (-1)	0.5 (-1)	5.52 ± 0.17
4	8 (1)	30 (1)	3 (-1)	0.05 (-1)	0.5 (-1)	7.2 (1)	1.5 (-1)	0.5 (1)	2 (1)	17.52 ± 0.33
5	8 (1)	5 (-1)	3 (-1)	0.05 (-1)	2.0 (1)	3.6 (-1)	3.0 (1)	0.5 (1)	0.5 (-1)	4.17 ± 0.26
6	3 (-1)	5 (-1)	3 (-1)	0.20 (1)	0.5 (-1)	7.2 (1)	3.0 (1)	0.1 (-1)	2 (1)	3.94 ± 0.20
7	3 (-1)	30 (1)	3 (-1)	0.20 (1)	2.0 (1)	3.6 (-1)	3.0 (1)	0.5 (1)	2 (1)	8.48 ± 0.17
8	8 (1)	5 (-1)	8 (1)	0.20 (1)	2.0 (1)	3.6 (-1)	1.5 (-1)	0.1 (-1)	2 (1)	7.93 ± 0.28
9	3 (-1)	30 (1)	8 (1)	0.20 (1)	0.5 (-1)	3.6 (-1)	1.5 (-1)	0.5 (1)	0.5 (-1)	3.51 ± 0.09
10	8 (1)	5 (-1)	8 (1)	0.20 (1)	0.5 (-1)	7.2 (1)	3.0 (1)	0.5 (1)	0.5 (-1)	4.62 ± 0.13
11	3 (-1)	5 (-1)	3 (-1)	0.05 (-1)	0.5 (-1)	3.6 (-1)	1.5 (-1)	0.1 (-1)	0.5 (-1)	2.91 ± 0.15
12	8 (1)	30 (1)	8 (1)	0.05 (-1)	0.5 (-1)	3.6 (-1)	3.0 (1)	0.1 (-1)	2 (1)	17.74 ± 0.23

Note: factors were shown as real values (coded levels). HPOPA values are shown as mean ± SD (n=3).

Supplementary Table S2. Experimental independent variables and their levels in the CCD (α = 1.682).

Variable factor	Symbol	Low axial-α	Low factorial-1	Center 0	High factorial+1	High axial+α
Glucose (g/L)	x_1	20	28.1	40	51.9	60
Peptone (g/L)	x_2	5	5.8	7	8.2	9
H_2O_2 (mL/100 mL)	x_3	0.33	0.60	1	1.40	1.67

Supplementary Table S3. CCD-based medium components for the production of HPOPA

Run	Factors			Response
	x_1 glucose (g/L)	x_2 peptone (g/L)	x_3 H_2O_2 (mL/100 mL)	HPOPA (g/L)
1	28.1 (-1)	5.8 (-1)	0.6 (-1)	17.15 ± 0.23
2	40 (0)	5 (-α)	1 (0)	17.09 ± 0.19
3	28.1 (-1)	5.8 (-1)	1.4 (1)	17.49 ± 0.34
4	40 (0)	7 (0)	1 (0)	19.05 ± 0.38
5	40 (0)	7 (0)	1.67 (α)	18.11 ± 0.05
6	28.1 (-1)	8.2 (1)	1.4 (1)	18.13 ± 0.29
7	51.9 (1)	8.2 (1)	0.6 (-1)	17.12 ± 0.17
8	40 (0)	7 (0)	0.33 (-α)	17.51 ± 0.28
9	40 (0)	7 (0)	1 (0)	19.37 ± 0.38
10	28.1 (-1)	8.2 (1)	0.6 (-1)	17.72 ± 0.22
11	51.9 (1)	8.2 (1)	1.4 (1)	17.48 ± 0.34
12	51.9 (1)	5.8 (-1)	1.4(1)	16.94 ± 0.13
13	40 (0)	7 (0)	1 (0)	19.25 ± 0.25
14	60 (α)	7 (0)	1 (0)	16.66 ± 0.40
15	51.9 (1)	5.8 (-1)	0.6 (-1)	16.65 ± 0.11
16	40 (0)	7 (0)	1 (0)	19.15 ± 0.33
17	40 (0)	9 (α)	1 (0)	17.84 ± 0.19
18	20 (α)	7 (0)	1 (0)	16.63 ± 0.26
19	40 (0)	7 (0)	1(0)	18.90 ± 0.21
20	40 (0)	7 (0)	1(0)	19.23 ± 0.36

Note: factors were shown as real values (coded levels). HPOPA values are shown as mean ± SD (n=3).

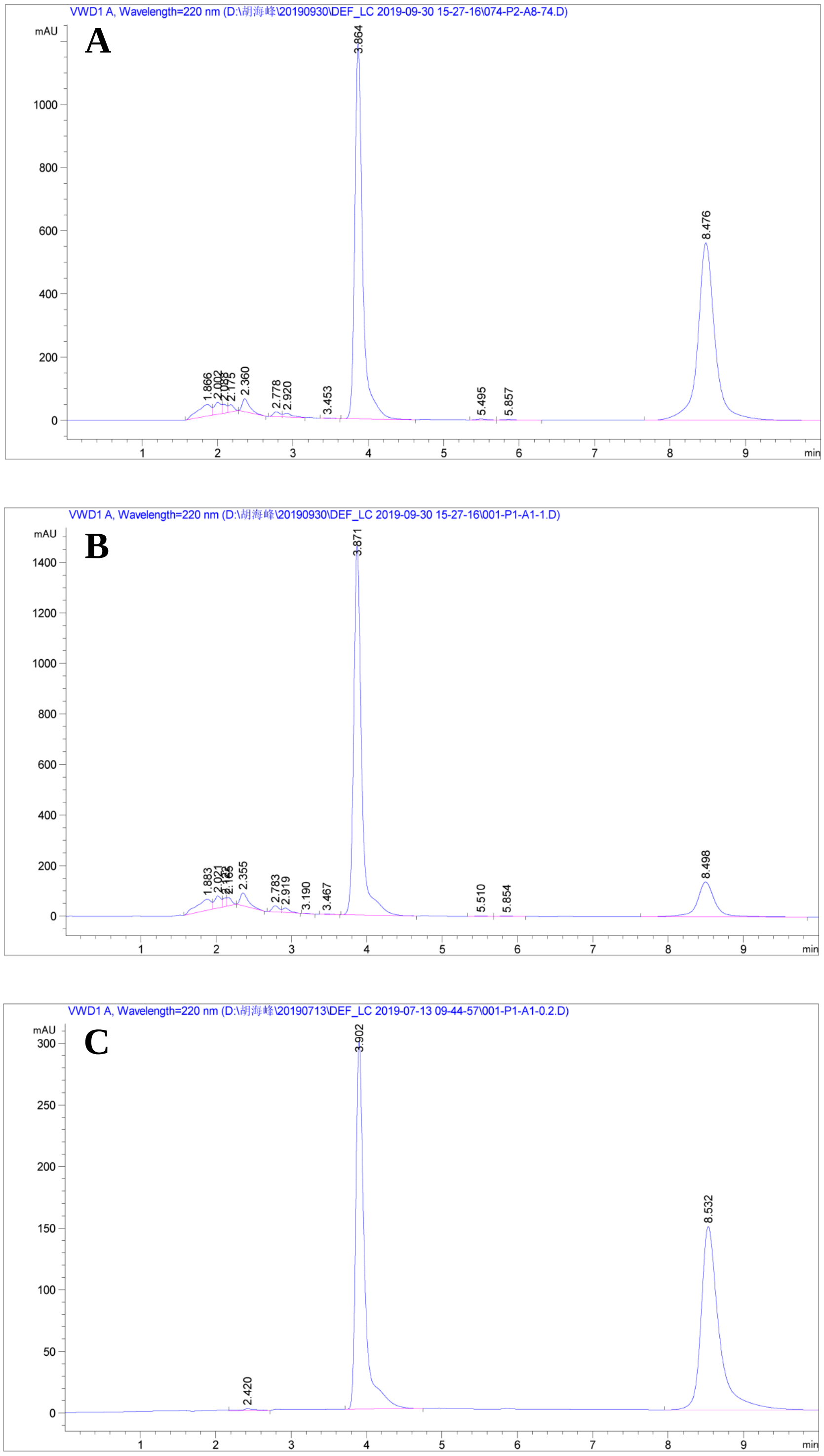

Supplementary Fig. S1. The HPLC chromatograms of HPOPA and POPA in the fermentation broth (diluted 20 times)

A, The original fermentation medium; B, The optimized fermentation medium; C, Standards of HPOPA (0.2 g/L) and POPA (0.2 g/L).

The retention time of HPOPA and POPA were 3.9 and 8.5 min, respectively.
